# Pembrolizumab-Induced Myocarditis and Pancreatitis in a Patient With Colon Cancer: A Case Report

**DOI:** 10.7759/cureus.26034

**Published:** 2022-06-17

**Authors:** Victor Delgado-Lazo, Wael Abdelmottaleb, Andrea Popescu-Martinez

**Affiliations:** 1 Medicine, Metropolitan Hospital Center, New York City, USA; 2 Oncology, Metropolitan Hospital Center, New York City, USA

**Keywords:** pembrolizumab, immune-checkpoint inhibitors, ca colon, medication-induced pancreatitis, immune therapy mediated myocarditis

## Abstract

We present a unique case of immune checkpoint inhibitor (ICI)-induced myocarditis and acute pancreatitis in a patient with metastatic colon cancer after seven cycles of pembrolizumab. A 43-year-old male with stage IV colon cancer on pembrolizumab presented with acute onset of heart failure with severely decreased ejection fraction (EF), conduction abnormalities, and normal coronary arteries on cardiac catheterization. He was started on high-dose steroids for grade 3 immune-related myocarditis. Four days later he presented with abdominal pain consistent with acute pancreatitis, likely related to the immune checkpoint inhibitors as well, Pembrolizumab was discontinued permanently. Pembrolizumab is currently used to treat many types of advanced cancers with promising results; thus, clinicians need to be aware of the multiple organs and systems that can be affected after using ICIs.

## Introduction

Immunotherapy including pembrolizumab has revolutionized cancer therapy in the past decade. Immune checkpoint inhibitors are monoclonal antibodies that block cytotoxic T lymphocyte-associated protein 4 (CTLA-4), programmed cell death protein-1 (PD-1), or programmed death-ligand 1 (PD-L1), each a key inhibitor of T cell activation and function. Thus, the activated immune response may lead to the development of multiple immune-related adverse events (IrAEs) affecting almost any organ system, from mild symptoms to severe life-threatening conditions.

Myocarditis associated with immune checkpoint inhibitors (ICI) is relatively rare with a wide incidence varying from 0.1% to 1%, it is often fulminant and lethal and frequently underdiagnosed and not well characterized due to limited data. Most cases occur early after the first dose, with a median time to onset of toxicity of 34 days after starting ICI [1]. The combination of ICI is the most well-established risk factor but diabetes, obesity, and obstructive sleep apnea (OSA) have also been reported. The clinical presentation of myocarditis includes signs of acute heart failure, including dyspnea, chest pain, conduction abnormalities [2], but surprisingly, nearly 50% of patients exhibit preserved systolic function [3]. Cardiac magnetic resonance (CMR) with late gadolinium enhancement (LGE) is the gold-standard non-invasive imaging test for diagnosis of myocarditis. However, LGE is present only in 48% of patients with ICI-induced myocarditis and, it seems to be time-dependent, Zhang et al. demonstrated that the presence of LGE increased from 21.6% when CMR was performed early to 72.0% when it was performed later after day 4 of admission [4]. 

ICI-related pancreatitis is mostly asymptomatic or mild, but fatal cases have been reported [5]. The incidence of pancreatitis in patients treated with a PD-1 inhibitor is around 0.94% [6]. Clinically it can present with typical symptoms of acute pancreatitis or it can be diagnosed incidentally with lipase elevations and imaging features of pancreatitis. CT findings are similar to traditional pancreatitis, with enlarged pancreas, surrounding fat stranding, and a decrease in attenuation [7]. Management depends on the severity of the IrAEs but generally includes the discontinuation of the ICI along with systemic steroids. Steroids are used for most of IrAEs, including myocarditis, pneumonitis, hepatitis, etc. However, there is limited data to support its use to treat immunotherapy-related pancreatitis [8]. 

## Case presentation

**Figure 1:**
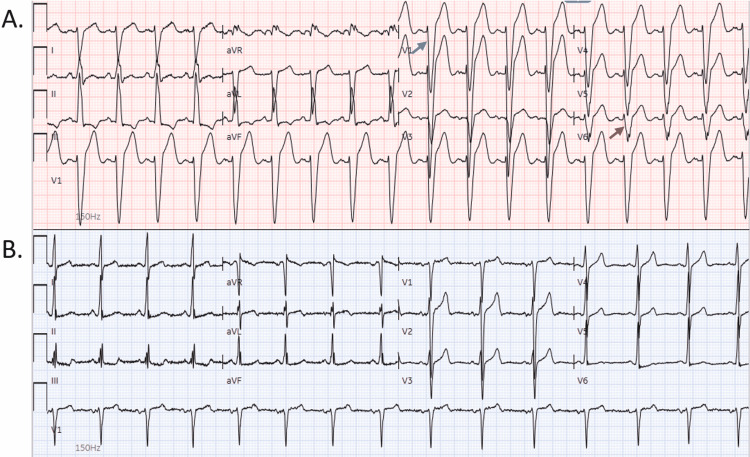
Electrocardiogram A. Electrocardiogram showing sinus tachycardia and a new left bundle branch block, characterized by prolonged QRS complexes (red arrow) and dominant S waves in V1 (blue arrow). B. Conduction abnormalities resolved after 3 days on steroids

A 43-year-old male with a past medical history of stage-IV colon cancer currently on monthly infusions of pembrolizumab for the past seven months, type 2 diabetes mellitus on metformin and insulin, and morbid obesity presented for acute onset of shortness of breath. Initial vitals signs were stable, Electrocardiogram (ECG) showed a new left bundle branch blockage (LBBB) (Figure 1), and he was noted to have elevated troponin T at 0.029 ng/mL, and computerized tomography (CT) angiography negative for pulmonary embolism. Further echocardiogram showed decreased ejection fraction (EF) of 15-20% with global hypokinesia. He was transferred for cardiac catheterization that revealed normal coronary arteries.

He was diagnosed with pembrolizumab-induced-myocarditis based on the rhythm abnormalities on the ECG, newly diagnosed heart failure, and global hypokinesia. He was started on methylprednisolone 125 mg IV (intravenous) daily with normalization of ECG abnormalities (Figure 1) and improvement of dyspnea. He also was started on furosemide, sacubitril-valsartan, metoprolol, and spironolactone for heart failure management. Cardiac magnetic resonance (CMR) performed on day five after admission showed mildly dilated left ventricle (LV) with global hypokinesis, but no abnormal late gadolinium enhancement and no areas of abnormal T2 signal in the LV myocardium. He was discharged on a long course of prednisone taper, sulfamethoxazole-trimethoprim for *Pneumocystis Jirovecii* prophylaxis, along with furosemide, sacubitril-valsartan, metoprolol, and spironolactone.

**Figure 2:**
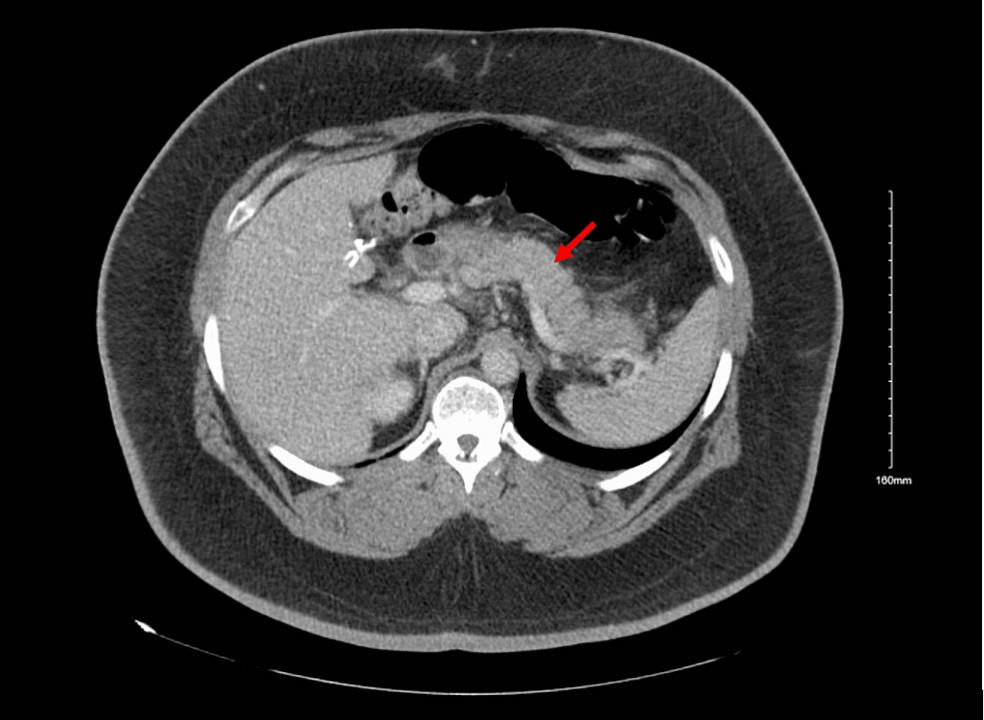
CT abdomen Computerized tomography of the abdomen showed peripancreatic stranding and haziness (arrow) suggestive of acute pancreatitis.

Four days after discharge, he was re-admitted for left upper quadrant and epigastric abdominal pain for two hours, intermittent, radiated to the chest and back. Vital signs were stable, laboratory remarkable for lipase of 588 U/L, CT abdomen showed peripancreatic stranding and haziness suggestive of pancreatitis (Figure 2). He was managed conservatively with analgesia and hydration. On further workup, an ultrasound of the abdomen showed diffuse hepatic steatosis but no stones, normal triglycerides at 98 mg/dL, and negative history of alcohol use. The pain improved and he continued on prednisone, along with sulfamethoxazole-trimethoprim, furosemide, sacubitril-valsartan, metoprolol, and spironolactone started the previous admission. Pembrolizumab was discontinued due to grade 3 ICI-induced myocarditis and grade 3 ICI-induced pancreatitis.

## Discussion

The diagnosis of myocarditis in patients treated with ICI is challenging due to the lack of specificity in the clinical presentation and given the multiple diagnostic tests required for differential diagnoses. Our patient presented with acute onset of heart failure, conduction abnormalities on the ECG, elevated troponins, and normal coronaries arteries on the cardiac catheterization. He showed significant improvement on steroids, making ICI-related myocarditis the most likely diagnosis. CMR is the gold-standard non-invasive imaging test for diagnosis, our patient did not show any abnormality to suggest inflammation or edema. Similarly, Zhang et al. reported that CMR might lack sensitivity to detect ICI-induced myocarditis compared with regular myocarditis [4]. In addition, the majority of cases of ICI-induced myocarditis present earlier after the first dose of pembrolizumab, we presented a case of myocarditis later after the seventh dose [4].

Clinicians should be aware of the increased risk for pancreatitis associated with immunotherapy but other potential causes such as cholelithiasis, alcohol, hypertriglyceridemia, and medications need to be investigated as well [9]. We ruled out the most common causes of pancreatitis in this case. However, it is difficult to establish a causal relationship in patients with medication-induced pancreatitis. Our patient was started on furosemide and sulfamethoxazole-trimethoprim during his first admission, both known causes of drug-induced pancreatitis [10]; however, based on the fact that the patient continued receiving both medications during the second admission without worsening symptoms of pancreatitis (negative rechallenge), let us conclude that furosemide and sulfamethoxazole-trimethoprim were less likely to be the cause of pancreatitis. Due to high suspicion of ICI-related pancreatitis, our patient continued on steroids previously started. Pembrolizumab was discontinued permanently due to the severity of adverse reactions.

Immune-related adverse events due to pembrolizumab may occur in multiple organs and systems, but not many case reports have shown multiple organs involved simultaneously. Suda et al. reported a simultaneous occurrence of autoimmune pancreatitis and sclerosing cholangitis, Ito et al. reported a case of multiple adverse events affecting the lungs, skin, and pituitary gland during pembrolizumab treatment [11,12]. To our knowledge, this is the first reported case of myocarditis and pancreatitis due to pembrolizumab. Our patient is currently being followed in the oncology clinic without any progression of malignancy, he also continues with the same heart failure medication regimen with mild improvement in the EF (35%) after a one-year follow-up.

## Conclusions

In summary, we have reported a unique case of ICI-induced myocarditis and acute pancreatitis in a patient with metastatic colon cancer after seven cycles of pembrolizumab. Pembrolizumab is currently being used to treat multiple types of cancers and clinicians need to be aware of the multiple organs and systems that can be affected after using ICIs.
